# Postural ergonomics and work-related musculoskeletal disorders in neurosurgery: lessons from an international survey

**DOI:** 10.1007/s00701-021-04722-5

**Published:** 2021-02-17

**Authors:** Georgios Mavrovounis, Torstein R. Meling, Jesus Lafuente, Konstantinos N. Fountas, Andreas K. Demetriades

**Affiliations:** 1 Department of Neurosurgery, Faculty of Medicine, University of Thessaly, Larisa, Greece; 2 Division of Neurosurgery, Department of Clinical Neurosciences, Geneva University Hospitals, Geneva, Switzerland; 3 Faculty of Medicine, University of Geneva, Geneva, Switzerland; 4 Spine Center, Hospital Del Mar, Barcelona, Spain; 5 Department of Neurosurgery, New Royal Infirmary, Edinburgh, UK

**Keywords:** Postural ergonomics, Ergonomics, Work-related musculoskeletal disorders, WMSDs, Questionnaire, Neurosurgery

## Abstract

**Background:**

Work-related musculoskeletal disorders (WMSDs) affect a significant percentage of the neurosurgical workforce. The aim of the current questionnaire-based study was to examine the prevalence of WMSDs amongst neurosurgeons, identify risk factors, and study the views of neurosurgeons regarding ergonomics.

**Methods:**

From June to August 2020, members of the “European Association of Neurosurgical Societies,” the “*Neurosurgery Research Listserv*,*”* and the “Latin American Federation of Neurosurgical Societies” were asked to complete an electronic questionnaire on the topics of WMSDs and ergonomics.

**Results:**

A total of 409 neurosurgeons responded to the survey, with a 4.7 male to female ratio. Most of the surgeons worked in Europe (76.9%) in academic public hospitals. The vast majority of the participants (87.9%) had experienced WMSDs, mainly affecting the shoulder, neck, and back muscles. The most common operations performed by the participants were “Craniotomy for convexity/intrinsic tumors” (24.1%) and “Open lumbar basic spine” (24.1%). Neurosurgeons agreed that ergonomics is an underexposed area in the neurosurgical field (84.8%) and that more resources should be spend (87.3%) and training curricula changes should be made (78.3%) in order to alleviate the burden of WMSDs on neurosurgeons. Univariate analysis did not reveal any associations between the development of WMSDs and age, gender, tenure, average duration of operation, operating time per week, type of operation, and surgical approach.

**Conclusions:**

The problem of WMSDs ought to be more closely addressed and managed by the neurosurgical community. More studies ought to be designed to investigate specific ergonomic parameters in order to formulate practice recommendations.

**Supplementary Information:**

The online version contains supplementary material available at 10.1007/s00701-021-04722-5.

## Introduction

In recent years, the occupational mental burden and its effects on physicians’ health, namely, burnout, have been given a lot of attention and have been extensively studied [7, 25]. On the contrary, albeit work-related physical burden is also prominent in the medical profession, especially amongst surgeons, it is not as widely studied and addressed.

Work-related musculoskeletal disorders (WMSDs) are injuries that affect various elements of the musculoskeletal system, such as the muscles, the tendons, the nerves, and the joints [12]. Their prevalence amongst surgeons is reported to be between 20 and 70% [2, 15], with the most commonly affected muscle groups being those of the neck, shoulders, and lower back [24]. WMSDs in surgeons can lead to numerous disease processes such as carpal tunnel syndrome, lumbar/cervical radiculopathy, varicose veins, and rotator cuff disease [6, 9, 17].

Such injuries do not solely have an effect on the surgeons’ ability to operate, but also have a significant impact on patient care as well. WMSD is the number one cause of absenteeism amongst healthcare workers, thus indirectly decreasing the healthcare workforce and consequently increasing the patient waiting time [42]. More importantly, WMSDs have been shown to reduce dexterity, range of motion, grip strength, and proprioception, with a direct impact on optimal patient’s care [30, 37, 40].

The International Ergonomics Association Council defines ergonomics as “the scientific discipline concerned with the understanding of interactions among humans and other elements of a system, and the profession that applies theory, principles, data, and methods to design in order to optimize human well-being and overall system performance [21].” It has been proposed that ergonomics can facilitate surgeons in the process of altering their everyday practice to alleviate the physical stressors that cause WMSDs and improve their general well-being [15]. Although several studies have looked into the subject of WMSDs and postural ergonomics in relation to the practice of general, orthopedic, and gynecologic surgery [6, 10, 27], the neurosurgery-related literature is limited.

The aim of the current questionnaire-based cross-sectional study was to examine the prevalence of WMSDs amongst neurosurgeons, identify possible risk factors in developing such disorders, and investigate neurosurgeons’ views and attitudes regarding postural ergonomics.

## Materials and methods

The present study constitutes a questionnaire-based, cross-sectional survey developed based on previously published literature on the subject of postural ergonomics in the surgical field [6, 10, 19, 20]. The “Google Forms” online platform (Google, Inc.) was used to distribute an electronic questionnaire to the members of the *European Association of Neurosurgical Societies* (EANS), utilizing the EANS mailing list (c.2000), Twitter and Facebook account between June 3, 2020, and August 11, 2020. Furthermore, the questionnaire was distributed through email and Facebook posts to the members of neurosurgery-related groups [e.g., “*Neurosurgery Research Listserv*” (8000 members)], and the members of the *Latin American Federation of Neurosurgical Societies* (FLANC) (c.2000). Reminder e-mails were sent 2 and 4 weeks after initial distribution to increase the response rate. The survey did not collect any data through which the participants could be personally identified.

The participants were asked to answer 38–49 questions (based on their answers) covering four major areas of interest, namely, (1) demographics and general information, (2) health-related information focusing on the musculoskeletal system, (3) procedure-specific information, and (4) personal views and attitudes regarding ergonomics.

### Statistical analysis

All statistical analysis calculations were performed using the GraphPad Prism (version 8.4.0 for MacOS, GraphPad Software, San Diego, California USA, www.graphpad.com). Categorical variables were analyzed and tested for statistical significance by the use of the Fisher’s exact and χ^2^ test, as appropriate. The statistical significance threshold was set at *p* = 0.05.

## Results

### Participants’ characteristics

A total of 409 neurosurgeons responded to the distributed questionnaire, with a 4.7 male to female ratio. Of those, 63 (15.5%) were trainees, 17 (4.2%) were fellows, and 327 (80.3%) were specialists/consultants. The vast majority (*N* = 296, 73.8%) of the responders worked in Europe, while 313 (76.9%) practiced medicine in Academic Public Hospitals (APH).

Regarding their surgical caseload, 126 surgeons (31.1%) reported that they mainly perform spine surgery, 181 (44.7%) mainly cranial, and 98 (24.2%) participants mentioned that they perform spine and cranial surgery equally often. Most of the responders (*N* =262, 64.4%) reported that they perform between 100 and 300 operations per year. The demographic characteristics and general information of the responders are presented in Table 1.

**Table 1:** Table presenting the demographic data of the participants

Question	n (% or SD)
Gender Male Female	409 337 (82.4) 72 (17.6)
Age, years < 35 35–45 45–54 55–64 ≥ 65	409 77 (18.8) 140 (34.2) 103 (25.2) 65 (15.9) 24 (5.9)
Mean BMI	
Female Male	22.5 kg/m^2^ (3.25) 26.7 kg/m^2^ (4.05)
Dominant hand Right Left Both	408 354 (86.8) 21 (5.1) 33 (8.1)
Position Trainee Fellow Specialist	407 63 (15.5) 17 (4.2) 327 (80.3)
Glove size 5.5–6.5 7–8 > 8	407 51 (12.5) 334 (82) 22 (5.4)
Tenure, years ≤ 15 > 15	404 199 (49.3) 205 (50.7)
Continent Europe Asia South America North America Africa Australasia	401 296 (73.8) 52 (13) 23 (5.7) 17 (4.2) 11 (2.7) 2 (0.5)
Practice type APH NAPH IPP GPP More than one APH+NAPH APH+GPP APH+IPP APH+GPP+IPP APH+NAPH+IPP+GPP APH+NAPH+IPP NAPH+IPP NAPH+GPP NAPH+IPP+GPP IPP+GPP	407 249 (61.2) 43 (10.6) 17 (4.2) 26 (6.4) 72 (17.7) 11 (15.3) 16 (22.2) 30 (41.7) 4 (5.6) 1 (1.4) 2 (2.8) 3 (4.2) 1 (1.4) 1 (1.4) 3 (4.2)
Scope of practice Pediatric neurosurgery Spine Neuro-oncology Neurovascular Skull base Pituitary Peripheral nerves Epilepsy Functional neurosurgery Neurotrauma Not focusing in a specific area	407 82 (20.1) 216 (53.1) 234 (57.5) 106 (26) 156 (38.3) 104 (25.6) 51 (12.6) 27 (6.6) 45 (11.1) 146 (35.9) 49 (12)
Surgical caseload Mainly spine Equal spine and cranial Mainly cranial	405 126 (31.1) 98 (24.2) 181 (44.7)
Operations/year < 100 100–300 > 300	407 74 (18.2) 262 (64.4) 71 (17.4)
Average duration of operation, hours < 3 > 3	406 245 (60.2) 161 (39.8)
Operating time/week (hands-on time), hours ≤ 10 11–19 ≥ 20	403 165 (40.9) 136 (33.7) 102 (25.3)

*SD* standard deviation, *BMI* body mass index, *APH* academic public hospital, *NAPH* non-academic public hospital, *IPP* individual private practice, *GPP* group private practice

### Health-related information

**Fig. 1:**
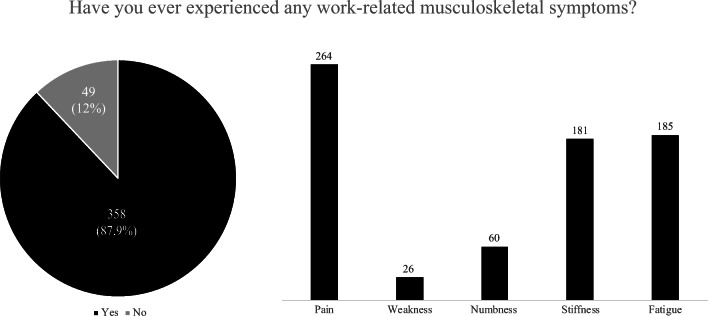
Figure illustrating the prevalence of work-related musculoskeletal disorders amongst the participants and the type of symptoms reported

**Fig. 2:**
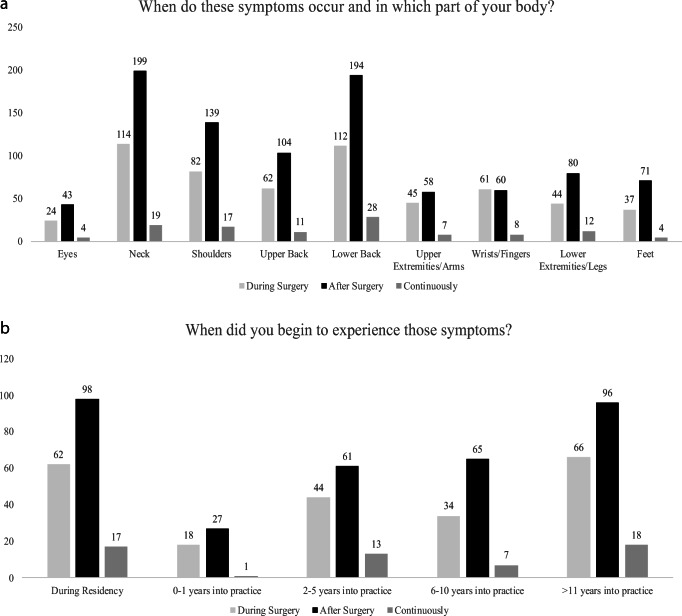
Figure illustrating the answers to the questions about (**a**) the part of the body that the participants experienced work-related musculoskeletal symptoms and (**b**) the time in their careers that they started to experience those symptoms

Regarding WMSDs, 358 (87.9%) of the participants reported that they have experienced musculoskeletal symptoms related to their work at least once in their career, predominantly pain (*N* = 264, 73.7%) (Fig. 1). Neck, shoulder and back were the most commonly symptomatic body parts, mainly affected after performing surgery (Fig. 2a). It is important to note that a substantial percentage of the responders (*N* = 98, 27.4%) started experiencing WMSDs after performing surgery while still in residency (Fig. 2b). This may indicate that early training regarding ergonomics and WMSDs are needed in order to educate young trainees on learning how to operate efficiently and ergonomically, something that more experienced neurosurgeons have learned through exposure.

Out of those with symptoms, only 30 (8.4%) had to decrease their case volume; however, 215 (60.4%) have sought some kind of treatment for their symptoms. Interestingly, only 28 (7.85%) participants reported that they had taken time-off work due to their symptoms. Table 2 summarizes the health-related data of the participants.

**Table 2:** Table presenting the health-related information of the participants

Question	*n* (%)
Rate your overall health	407
Excellent	111 (27.3)
Very good	176(43.2)
Good	107 (26.3)
Fair	12 (2.9)
Poor	1 (0.2)
Exercise time/week, hours	406
0	86 (21.2)
1–4	233 (57.4)
> 4	87 (1.4)
Experienced non-work-related MSK injury	406
Yes	173 (42.6)
Non-specified	12 (7)
Neck	9 (5.2)
Chest	3 (1.7)
Head	1 (0.6)
Lower extremities	61 (35.3)
Feet	29 (16.8)
Upper extremities	44 (25.4)
Hands	12 (6.9)
Back	54 (31.2)
No	233 (57.4)
Experienced work-related MSK symptoms	407
Yes	358 (87.9)
Pain	264 (73.7)
Weakness	26 (7.3)
Numbness	60 (16.8)
Stiffness	181 (50.6)
Fatigue	185 (51.7)
No	49 (12)
Duration of work-related MSK symptoms, years	354
< 3	177 (50)
3–6	96 (26.8)
6–9	32 (9)
> 9	50 (14.1)
Procedures that are more likely to cause pain/discomfort	
All long procedures	30
Procedures involving bone work	2
Spine	110
Skull base	26
Craniotomy (non-specified)	9
Endoscopy	1
Procedures involving the use of microscope	8
Oncology	14
Vascular	4
Peripheral nerve	1
Pineal + posterior fossa lesions (patient in the sitting position)	24
TTH	5
Skull deformities	1
Decreased case-volume due to MSK symptoms	357
Yes	31 (8.7)
Non-specified	13 (41.9)
Skull-base	2 (6.5)
Spine	7 (22.6)
Vascular	1 (3.2)
Endoscopic	2 (6.5)
Oncology	2 (6.5)
Peripheral	3 (9.7)
Cranial (non-specified)	1 (3.2)
No	326 (91.3)
Continue working despite the MSK symptoms	356
Yes	353 (99.2)
No	3 (0.8)
Sick leave	357
Yes [Mean time, days (SD): 28 (45.7), information for 28/29]	29 (8.1)
No	329 (91.9)
Sought treatment for the MSK symptoms	356
Yes	215 (60.4)
Analgesics	77 (35.8)
Surgery	5 (2.3)
Physical therapy	57 (26.5)
Analgesics + physical therapy	63 (29.3)
Analgesics + surgery	3 (1.4)
Physical therapy + surgery	2 (0.9)
Analgesics + physical therapy + surgery	8 (3.7)
No	141 (39.6)
Specify the type of surgery	18
Carpal tunnel release	1 (5.6)
Cervical discectomy	6 (33.3)
Lumbar discectomy	5 (27.8)
Discectomy (non-specified)	2 (11.1)
Foraminotomy/laminectomy (non-specified)	2 (11.1)
Knee arthroplasty + other orthopedic	2 (11.1)
MSK symptoms resolved after treatment	211
Yes	80 (37.9)
No	18 (8.5)
Partially	93 (44.1)
Only temporarily	48 (22.7)

*MSK* musculoskeletal, *TTH* trans-nasal trans-sphenoidal hypophysectomy, *SD* standard deviation

### Procedure-specific information

**Fig. 3:**
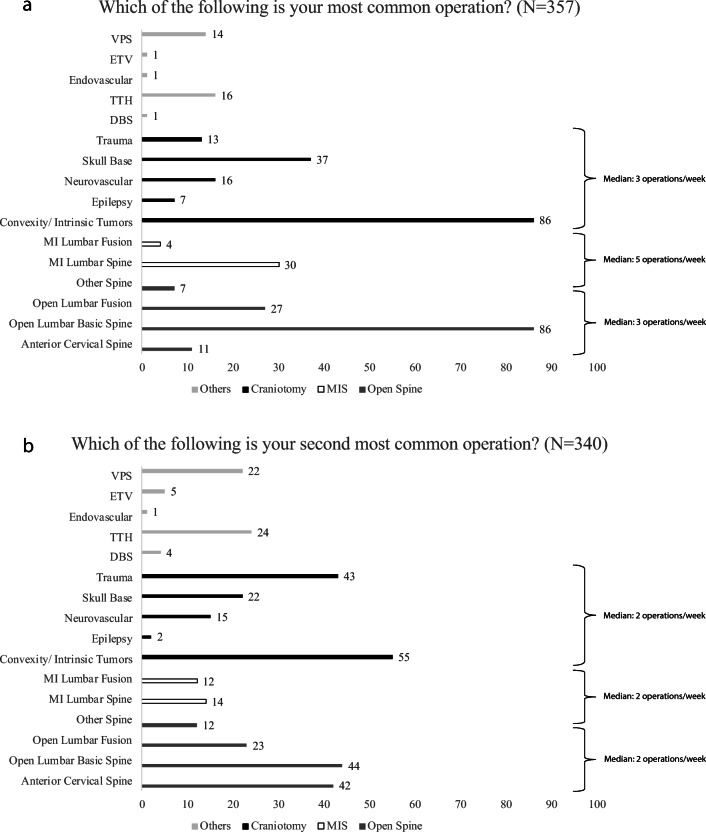
Figure presenting (**a**) the most common operation and (**b**) the second most common operation as reported by the participants

When asked to choose the procedure, they perform most commonly from a list of routine neurosurgical procedures, 159/357 (44.5%) answered that they perform “craniotomies” and 165/357 (46.2%) that they perform “spinal surgery”. More specifically, 24.1% (*N* = 86) reported that they perform “craniotomy for convexity/intrinsic tumors.” The same number of responders (*N* = 86, 24.1%) reported that they perform “open lumbar basic spine” procedures. “Craniotomy for skull base diseases” (*N* = 37, 10.4%) and “minimally invasive lumbar spine” (*N* = 30, 8.4%) are some other procedures that were also reported by the participants (Fig. 3a).

Following a similar trend, when asked about their second most common operation, 137/340 (40.3%) answered that they perform “craniotomies” and 147/340 (43.2%) that they perform “spinal surgery.” More specifically, 16.2% (*N* = 55) reported that they perform “craniotomy for convexity/intrinsic tumors” followed by “open lumbar basic spine” (*N* = 44, 12.9%), “craniotomy for trauma” (*N* = 43/340, 12.6%), and “anterior cervical spine” operations (*N* = 42/340, 12.4%) (Fig. 3b).

Information regarding intraoperative practice for the most commonly mentioned types of procedures are presented in Appendix A (Tables 4 and 5) as Electronic Supplementary Materials. Notably, very few surgeons answered that they routinely use “chairs with back and neck support” [(*N* = 12/354) 3.4%, during most common operation; (*N* = 15/322) 4.7%, during second most common operation], and “arm supports” [(*N* = 30/354) 8.5%, during most common operation (N=20/322); and 6.2%, during second most common operation]. Appendix B (Tables 6 – 9) as Electronic Supplementary Materials presents information regarding the intraoperative practice of spine surgeons on the use of lead apron for fluoroscopy and navigation. Regarding open-spine surgery, the majority of surgeons reported that they are using navigation and/or fluoroscopy [(*N* = 118/131) 90%, during most common operation and (*N* = 106/121) 87.6%, during second most common operation]. A similar trend was found in those performing minimally invasive spinal surgery [(*N* = 33/34) 97.1%, during most common operation and (*N* = 22/24) 91.7%, during second most common operation]. Most surgeons that are routinely using fluoroscopy for their most common operation reported that they use a protective lead apron (open spine: 70/85, 82.4%, minimally invasive spine: 14/17, 82.4%). Notably, neither open [(fluoroscopy: *N* = 106/111, 95.5%; navigation: *N* = 30/33, 91%) during most common operation, (fluoroscopy: N=94/100, 94%; navigation: N=18/19, 94.7%) during second most common operation], nor minimally open [(fluoroscopy: N=30/32, 93.8%; navigation: N=15/16, 93.8%) during most common operation, (fluoroscopy: N=19/21, 90.5%; navigation: N=10/10, 100%) during second most common operation] spine surgeons are wearing eye protection when using navigation and/or fluoroscopy.

**Fig. 4:**
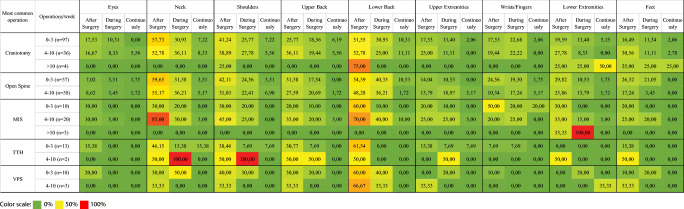
Figure presenting a heat-map that depicts the physical burden of the most commonly mentioned types of procedures on the various parts of the body, based on the number of operations per week. Notably, regardless of the procedure, the most commonly affected parts of the body were the neck, shoulders, and lower back

Figure 4 demonstrates the physical burden of the most commonly mentioned types of procedures on the various parts of the body, based on the number of operations performed per week. Regardless of the type of the procedure, the most commonly affected parts of the body were the neck, shoulders, and lower back. The least affected areas of the body were the eyes and the wrists/fingers. Most surgeons complained of pain mainly after and during surgery, and it is important to note that very few reported that they are experiencing symptoms on a continuous basis.

### Views and attitudes on postural ergonomics

The overwhelming majority of the responders believe that the physical burden on healthcare practitioners is an underexposed area in medicine (*N* = 320/400, 80%). Similarly, they also believe that postural ergonomics, in particular, is an underexposed area in the neurosurgical field (*N* = 340/401, 84.8%).

Evidently, 314/401 (78.3%) reported that changes should be made in the training curricula of trainees in order for them to receive education and training on the topic of surgical ergonomics. Furthermore, 349/400 (87.3%) believe that hospital management authorities should invest more resources in order to equip the operating rooms more ergonomically (Appendix C—Table 10) as Electronic Supplementary Materials.

### Univariate analysis

Table 3 presents an overview of the results of the univariate analysis based on experiencing WMSDs. No associations were found between the development of WMSDs and the age/gender of the responders, their tenure, and the average duration of their operations. Moreover, no associations were found regarding the time they spend operating per week, their most common operations (craniotomy vs spine), the use of lead protection while operating, and the surgical approach (open versus minimally invasive).

**Table 3:** Table summarizing the results of the univariate analysis.

Parameter	Univariate analysis		
	Symptoms	No symptoms	*p* value
Gender			0.17
Male	291	44	
Female	67	5	
Age, years			0.65
< 45	191	24	
≥ 45	167	25	
BMI, kg/m^2^			>0.99
< 25	171	23	
≥ 25	180	25	
Tenure, years			0.76
≤ 15	176	23	
> 15	179	26	
Surgical caseload			0.50
Mostly Cranial	162	19	
50–50%	83	15	
Mostly spine	111	15	
Operations/year			0.047
< 100	65	9	
100–300	232	10	
> 300	61	10	
Average duration of operation, hours			0.53
< 3	213	32	
> 3	145	17	
Operating time/week, hours			0.71
≤ 10	146	19	
11–19	122	14	
≥ 20	88	14	
Exercise time/week, hours			0.42
0	77	9	
1–4	207	26	
> 4	73	14	
Most common operation			0.52
Craniotomy ^a^	167	25	
Spine	163	19	
Most common operation			0.49
Skull base + TTH	53	6	
Other craniotomy ^b^	114	19	
Most common operation			0.08
Open spine	122	18	
MIS	41	1	
Second most common operation			0.39
Craniotomy ^a^	160	22	
Spine	152	15	
Second most common operation			0.75
Skull base + TTH	24	4	
Other craniotomy ^b^	136	18	
Second most common operation			0.31
Open spine	123	14	
MIS	32	1	
Use of lead protection ^c^			0.36
Yes	175	20	
No	183	29	

*BMI* body mass index, *TTH* trans-nasal trans-sphenoidal hypophysectomy, *MIS* minimally invasive spine

^a^Trauma, skull base, neurovascular, epilepsy, convexity/intrinsic tumors

^b^Trauma, neurovascular, epilepsy, convexity/intrinsic tumors

^c^First and second most common operations combined

Of note, a statistically significant larger number of participants in the 100–300 operations per year group reported WMSDs when compared with the > 300 operations per year group. Similarly with the training level, it is unclear whether this indicates that surgeons with a higher volume of cases learn to work more ergonomically.

## Discussion

### Summary

The present questionnaire-based study surveyed 409 neurosurgeons to assess the effect of WMSDs in the neurosurgical field. Our results reveal that WMSDs is a prevalent issue in the field, as more than 85% of the participants reported that they have previously experienced some musculoskeletal discomfort associated with work-related exposure. Complaints associated with the neck, the back and the shoulders were commonly mentioned by the responders, mainly occurring after performing surgery. A sizeable percentage of those who have experienced WMSDs have sought treatment, using analgesics and physical therapy. Most neurosurgeons reported that they believe that “ergonomics is an underexposed area in the neurosurgical field”, and that young neurosurgeons should be educated and trained on the subject while still in training. It is worth noting that our results hint that WMSDs start early in the course of a neurosurgeons’ career (even during residency) and that surgeons with a higher volume of operations may empirically learn to work more ergonomically. As a results, it could be beneficial for young trainees and specialists to attend courses designed by experts and senior neurosurgeons on the subject of WMSDs.

Our study did not reveal any associations between the development of WMSDs and any of the factors analyzed, maybe indicating that WMSDs are a global problem in neurosurgery irrespective of the gender, age, tenure, operating volume or approach of surgery (open versus minimally invasive) etc.

### Literature overview

In recent years, increased awareness of the physical burden of operating on surgeons has led to the publication of several studies investigating the subject amongst various surgical specialties [6, 19, 20]. Most of the authors conclude that WMSDs are an important problem in the surgical profession and advocate for further research on the field of postural ergonomics in surgery.

The literature pertaining to the field of postural ergonomics in neurosurgery is limited. Gadjradj et al. [19], in a recent survey amongst neurosurgeons, reported results similar to those of our present study. Of importance, they identified a tenure of more than 15 years to be associated with the development of WMSDs, specifically pain/discomfort, a result not replicated in our analysis. However, a previous study performed amongst spine surgeons [6] did not show any correlation between years of practice and WMSDs development.

### The gender factor

It has been previously reported, in studies among the general population and various occupations, that the prevalence of WMSDs is greater amidst women [11, 41, 43]. It has been proposed that the smaller body size and anthropometric measurements of females may lead to a higher workload when performing the same tasks as males [39]. Furthermore, several studies suggest that sex hormones (e.g., estrogens) affect pain perception and argue that lower estrogen levels during some phases of the menstrual cycle may lead women to report more symptoms than men [3, 4, 16]. Interestingly, our study did not find any gender-based differences in the prevalence of symptoms when comparing females versus males.

### Minimally invasive versus open surgery

The establishment of the concept of minimally invasive surgery and the implementation of minimally invasive techniques, especially in the fields of general and gynecological surgery, has fundamentally altered patient care [34]. However, minimally invasive procedures (e.g., laparoscopic and endoscopic) have been traditionally associated with increased WMSDs [2, 31]. Endoscopic procedures are frequently performed in neurosurgery and have been associated with upper limb and shoulder pain [26].

In the present study, minimally invasive spine (MIS) procedures did not seem to increase WMSDs when compared with open spine surgery. Furthermore, when skull base surgery and trans-nasal trans-sphenoidal hypophysectomy were compared with “other craniotomy” procedures, no statistically significant difference in WMSDs was identified.

When directly asked whether they believe that “Minimally invasive surgery leads to more physical discomfort than open surgery,” participants of both the present study and the study by Gadjradj et al. [19] were mostly neutral about their opinion (40.3% in our current study, 39.8% in Gadjradj et al.); opinions were divided amid the rest of the responders.

### Cranial versus spine surgery

In line with a general view in the neurosurgical community, several authors have reported that specialists who mostly perform spine surgical procedures suffer more from WMSDs than their colleagues who perform mostly cranial surgery [19, 26, 38]. Surgeons that perform mostly craniofacial surgery usually report musculoskeletal symptoms related to the upper limbs [32, 38], whereas spine surgeons also report neck and shoulder symptoms [6].

We, however, did not find any correlation between the type of procedure and WMSDs when we compared the prevalence of WMSDs between cranial and spinal neurosurgeons. This could reflect the universal nature of WMSDs in neurosurgery and the need for educating neurosurgeons in order to be aware and mindful of this occupational risk.

### Intraoperative routine and equipment

Prolonged standing periods have been previously associated with increased lower back, leg, and feet pain [36]. Several authors have suggested that a sitting position should be preferred for long tasks, such as microsurgical interventions and suturing [8, 22, 23]. However, the results of our study suggest that most surgeons spend the majority of their operating time in the standing position. In order to minimize physical burden, specific training courses and trainee education could focus on teaching young neurosurgeons to effectively operate while sitting, when appropriate.

In a study amongst surgeons performing vaginal surgery, chairs with round, flat seats, and back support were reported to be more comfortable than those with saddle-shaped seats and no back support [35]. Our results indicate that most neurosurgeons use a chair without back and neck support. This may indicate that operating rooms are not furnished with ergonomic equipment and that more careful planning and funds should be spent in that direction.

It has been previously reported that, although loupes offer several advantages such as portability and cost-effectiveness, procedures performed with them are associated with extreme neck angles and increased muscle workload [13, 44]. On the other hand, operating with the use of a microscope allows surgeons to maintain a neutral head position and offers a better view of the surgical field [13]. When available and appropriate, the microscope should be preferred to the loupes as it can increase surgeon’s comfort and make assisting and operating safer and easier.

Spine surgeons often use fluoroscopy-guided techniques to enable correct instrumentation and execution of procedures. In order to minimize radiation exposure, they usually wear lead aprons that can weigh up to 17 kg [1]. It has been reported that wearing a lead apron increases discomfort and fatigue, especially on the muscle groups of the back [1]. Although the majority of participants agree that wearing a lead apron increases physical discomfort, our univariate analysis did not reveal a statistically significant difference in WMSDs occurrence in spine surgeons that reported frequent lead apron usage.

### Future considerations

The field of postural ergonomics in surgery is becoming increasingly popular in recent years, leading to an increased effort by the surgical community to find solutions regarding the problem of WMSDs. It is important to educate trainees and young neurosurgeons to be mindful of the related occupational risks that they will be inevitably exposed to throughout their careers. This could be achieved by officially incorporating postural ergonomics education into the training curricula of neurosurgery residents and can also be facilitated by courses on specific topics organized by neurosurgical societies. In 2013, Franasiak et al. reported that after attending ergonometric training designed by an expert, 88% of their study participants (robotic surgeons) changed practice, with 84% reporting reduction in musculoskeletal strain [18]. Interestingly, another study showed that training in the Alexander technique, a method that is used to change and improve movement habits, resulted in improved posture and less discomfort amongst urological surgeons [33].

Furthermore, more studies focusing on postural ergonomics, surgical instrument design, and operating theater equipment should be designed to identify ideal ergonomics for neurosurgery. An interesting approach was used by researchers from the Mayo Clinic, USA, who used wearable sensor inertial measurement units to study the posture of surgeons while operating [28]. In recent years, the concept of intraoperative microbreaks has been studied in order to identify if microbreaks can result in less fatigue. In a 2013 study, Dorion and Darveau reported that 20-s-long intraoperative microbreaks every 20 min to stretch the neck and shoulders resulted in statistically significant less discomfort in all body areas of the study participants (general surgeons, neurosurgeons, head and neck surgeons, cardiac surgeons) [14].

### Limitations

The current study has some limitations that should be acknowledged. Firstly, recall bias is an important factor in all survey-based studies, and it is particularly important in studies like ours that ask participants to recall information regarding careers spanning more than 45 years in some cases [5]. Additionally, the number of responders in our study was limited when compared with the global (≈ 50,000 neurosurgeons) and even the European (≈ 11,000) neurosurgical workforce [29]. Finally, because of the design of our study (mainly focused on EANS members), the vast majority of responders practise in Europe, introducing selection bias. These limitations make careful interpretation of our results necessary.

## Conclusion

Postural ergonomics and WMSDs are important topics, which deserve more attention from the neurosurgical community, as a significant percentage of neurosurgeons has experienced WMSDs at some point throughout their career. Further research has to be conducted in order to shed more light on specific areas of interest, such as those of postural ergonomics and operating theater equipment. Trainees and young neurosurgeons ought to be educated on the subject and receive specific training, in order to adopt healthy attitudes and minimize WMSDs.

## Supplementary Information

Appendix 1 Tables presenting data regarding the intraoperative practice of the participants during their (Table 4) most commonly performed operation and their (Table 5) second most commonly performed operation. (DOCX 21 kb)

Appendix 2 Table presenting the intraoperative use of navigation and fluoroscopy. (DOCX 18 kb)

Appendix 3 Table presenting the views and attitudes of the participants regarding ergonomics (Table 10) (DOCX 15 kb)

## Abbreviations

WMSDs: Work-related musculoskeletal disorders
EANS: European Association of Neurological Societies
FLANC: Latin American Federation of Neurosurgical Societies
APH: Academic Public Hospitals
USA: United States of America

## Declarations

All procedures performed in studies involving human participants were in accordance with the ethical standards of the institutional and/or national research committee and with the 1964 Helsinki declaration and its later amendments or comparable ethical standards.
