# Supplementary material for: Postural ergonomics and work-related musculoskeletal disorders in neurosurgery: lessons from an international survey
Source: Acta Neurochir (Wien). 2021 Feb 17;163(6):1541–52. doi: 10.1007/s00701-021-04722-5 (PMC8116287; doi:10.1007/s00701-021-04722-5)
Supplement: Supplementary file 1 — Tables presenting data regarding the intraoperative practice of the participants during their (Table 4) most commonly performed operation and their (Table 5) second most commonly performed operation. (DOCX 21 kb) [file 701_2021_4722_MOESM1_ESM.docx]

| Question | Open Spine (n=131) | MIS (n=34) | Craniotomy (n=159) | TTH (n=16) | VPS (n=14) |
| --- | --- | --- | --- | --- | --- |
| Operate while sitting (for most of the operation) | 4 (3%) | 0 | 88 (55.3%) | 1 (6.3%) | 1 (7.1%) |
| Operate while standing (for most of the operation) | 124 (94.7%) | 34 (100%) | 73 (45.9%) | 15 (93.8%) | 13 (92.9%) |
| Use a chair with back and neck support | 0 | 0 | 11 (6.9%) | 0 | 1 (7.1%) |
| Use a chair without back and neck support | 9 (6.9%) | 0 | 55 (34.6%) | 1 (6.3%) | 3 (21.4%) |
| Routinely use arm supports | 1 (0.8%) | 1 (2.9%) | 28 (17.6%) | 0 | 0 |
| Routinely stand on a footstool (for added height) | 7 (5.3%) | 4 (11.8%) | 4 (2.5%) | 0 | 1 (7.1%) |
| Routinely wear loupes | 16 (12.2%) | 4 (11.8%) | 14 (8.8%) | - | 1 (7.1%) |
| Routinely wear a head light source | 12 (9.2%) | 5 (14.7%) | 6 (3.8%) | - | 0 |
| Routinely use a microscope | 76 (58%) | 22 (64.7%) | 127 (79.9%) | - | - |
| Use foot pedals | 60 (45.8%) | 18 (52.9%) | 84 (52.8%) | 5 (31.3%) | - |
| Gaze upwards through the microscope | 7 (5.3%) | 1 (2.9%) | 22 (13.8%) | - | - |
| Gaze straight ahead through the microscope | 49 (37.4%) | 18 (52.9%) | 75 (47.2%) | - | - |
| Gaze downwards through the microscope | 23 (17.6%) | 4 (11.8%) | 46 (28.9%) | - | - |
| Routinely use power tools (drill) | 56 (42.7%) | 16 (47.1%) | 115 (72.3%) | 6 (37.5%) | 3 (21.4%) |
| Place the endoscope monitor above your eye level | - | - | - | 0 | - |
| Place the endoscope monitor in line with your eye level | - | - | - | 10 (62.5%) | - |
| Place the endoscope monitor below your eye level | - | - | - | 1 (6.3%) | - |
| Operate with your neck rotated towards the endoscope monitor | - | - | - | 4 (25%) | - |

**Appendix A**

Appendix Table 4

Abbreviations. MIS: Minimally Invasive Spine, TTH: Trans-nasal Trans-sphenoidal Hypophysectomy, VPS: Ventriculoperitoneal Shunt

| Question | Open Spine (n=121) | MIS (n=26) | Craniotomy (n=137) | TTH (n=16) | VPS (n=22) |
| --- | --- | --- | --- | --- | --- |
| Operate while sitting (for most of the operation) | 6 (5%) | 1 (3.8%) | 77 (56.2%) | 1 (6.3%) | 2 (9.1%) |
| Operate while standing (for most of the operation) | 111 (91.7%) | 24 (92.3%) | 62 (45.3%) | 15 (93.8%) | 20 (90.9%) |
| Use a chair with back and neck support | 0 | 0 | 15 (10.9%) | 0 | 0 |
| Use a chair without back and neck support | 1 (0.8%) | 0 | 37 (27%) | 1 (6.3%) | 2 (9.1%) |
| Routinely use arm supports | 0 | 0 | 20 (14.6%) | 0 | 0 |
| Routinely stand on a footstool (for added height) | 10 (8.3%) | 3 (11.5%) | 1 (0.7%) | 0 | 0 |
| Routinely wear loupes | 15 (12.4%) | 5 (19.2%) | 14 (10.2%) | - | 1 (4.5%) |
| Routinely wear a head light source | 11 (9.1%) | 1 (3.8%) | 6 (4.4%) | - | 1 (4.5%) |
| Routinely use a microscope | 73 (60.3%) | 19 (73.1%) | 85 (62%) | - | - |
| Use foot pedals | 57 (47.1%) | 18 (69.2%) | 66 (48.2%) | 5 (31.3%) | - |
| Gaze upwards through the microscope | 5 (4.1%) | 0 | 13 (9.5%) | - | - |
| Gaze straight ahead through the microscope | 42 (34.7%) | 14 (53.8%) | 54 (39.4%) | - | - |
| Gaze downwards through the microscope | 29 (24%) | 4 (15.4%) | 22 (16.1%) | - | - |
| Routinely use power tools (drill) | 48 (39.7%) | 13 (50%) | 72 (52.6%) | 6 (37.6%) | 14 (63.6%) |
| Place the endoscope monitor above your eye level | - | - | - | 0 | - |
| Place the endoscope monitor in line with your eye level | - | - | - | 9 (56.3%) | - |
| Place the endoscope monitor below your eye level | - | - | - | 1 (6.3%) | - |
| Operate with your neck rotated towards the endoscope monitor | - | - | - | 4 (25%) | - |

Appendix Table 5

Abbreviations. MIS: Minimally Invasive Spine, TTH: Trans-nasal Trans-sphenoidal Hypophysectomy, VPS: Ventriculoperitoneal Shunt
