# Supplementary material for: Postural ergonomics and work-related musculoskeletal disorders in neurosurgery: lessons from an international survey
Source: Acta Neurochir (Wien). 2021 Feb 17;163(6):1541–52. doi: 10.1007/s00701-021-04722-5 (PMC8116287; doi:10.1007/s00701-021-04722-5)
Supplement: Supplementary file 2 — Table presenting the intraoperative use of navigation and fluoroscopy. (DOCX 18 kb) [file 701_2021_4722_MOESM2_ESM.docx]

| Routinely use navigation + fluoroscopy (N=131) | Lead apron [n (mean hours/week)] | | | Eye protection (n) | | Thyroid protection (n) | |
| --- | --- | --- | --- | --- | --- | --- | --- |
|  | One-piece | Two-piece | No | Yes | No | Yes | No |
| Navigation only: 7 | 2 (3.5) | 2 (4.5) | 3 | 0 | 7 | 4 | 3 |
| Fluoroscopy only: 85 | 50 (5.6) | 20 (6) | 15 | 3 | 82 | 36 | 49 |
| Both: 26 | Nav: 5 (6.6)  Fl: 12 (5.6) | Nav: 4 (6.25)  Fl: 12 (4.4) | Nav: 17  Fl: 2 | Nav: 3  Fl: 2 | Nav: 23  Fl: 24 | Nav: 9  Fl: 20 | Nav: 17  Fl: 6 |
| None: 13 | - | - | - | - | - |  | - |

**Appendix B**

Appendix Table 6

Abbreviations. Nav: Navigation, Fl: Fluoroscopy

Appendix Table 7

Abbreviations. Nav: Navigation, Fl: Fluoroscopy

| Routinely use navigation + fluoroscopy (N=34) | Lead apron [n (mean hours/week)] | | | Eye protection (n) | | Thyroid protection (n) | |
| --- | --- | --- | --- | --- | --- | --- | --- |
|  | One-piece | Two-piece | No | Yes | No | Yes | No |
| Navigation only: 1 | 0 | 0 | 1 | 0 | 1 | 0 | 1 |
| Fluoroscopy only: 17 | 10 (6.1) | 4 (5) | 3 | 1 | 16 | 8 | 9 |
| Both: 15 | Nav: 7 (6.3)  Fl: 7 (6) | Nav: 1 (no info)  Fl: 5 (1) | Nav: 7  Fl: 3 | Nav: 1  Fl: 1 | Nav: 14  Fl: 14 | Nav: 4  Fl: 8 | Nav: 11  Fl: 7 |
| None: 1 | - | - | - | - | - | - | - |

Appendix Table 8

Abbreviations. Nav: Navigation, Fl: Fluoroscopy

| Routinely use navigation + fluoroscopy (N=121) | Lead apron [n (mean hours/week)] | | | Eye protection (n) | | Thyroid protection (n) | |
| --- | --- | --- | --- | --- | --- | --- | --- |
|  | One-piece | Two-piece | No | Yes | No | Yes | No |
| Navigation only: 6 | 1 (1) | 1 (8) | 4 | 1 | 5 | 3 | 3 |
| Fluoroscopy only: 87 | 20 (4.5) | 12 (3.8) | 55 | 11 | 82 | 41 | 46 |
| Both: 13 | Nav: 4 (10)  Fl: 5 (4.5) | Nav: 2 (4.25)  Fl: 5 (4) | Nav: 7  Fl: 3 | Nav: 0  Fl: 1 | Nav: 13  Fl: 12 | Nav: 4  Fl: 10 | Nav: 9  Fl: 3 |
| None: 15 | - | - | - | - | - |  | - |

Appendix Table 9

Abbreviations. Nav: Navigation, Fl: Fluoroscopy

| Routinely use navigation + fluoroscopy (N=26) | Lead apron [n (mean hours/week)] | | | Eye protection (n) | | Thyroid protection (n) | |
| --- | --- | --- | --- | --- | --- | --- | --- |
|  | One-piece | Two-piece | No | Yes | No | Yes | No |
| Navigation only: 3 | 0 | 0 | 3 | 0 | 3 | 0 | 3 |
| Fluoroscopy only: 14 | 9 (5.25) | 4 (3.5) | 1 | 2 | 12 | 9 | 5 |
| Both: 7 | Nav: 3 (4)  Fl: 4 (2.5) | Nav: 1 (no info)  Fl: 3 (1) | Nav: 3  Fl: 0 | Nav: 0  Fl: 0 | Nav: 7  Fl: 7 | Nav: 1  Fl: 3 | Nav: 6  Fl: 4 |
| None: 2 | - | - | - | - | - | - | - |
