# Supplementary material for: Postural ergonomics and work-related musculoskeletal disorders in neurosurgery: lessons from an international survey
Source: Acta Neurochir (Wien). 2021 Feb 17;163(6):1541–52. doi: 10.1007/s00701-021-04722-5 (PMC8116287; doi:10.1007/s00701-021-04722-5)
Supplement: Supplementary file 3 — Table presenting the views and attitudes of the participants regarding ergonomics (Table 10) (DOCX 15 kb) [file 701_2021_4722_MOESM3_ESM.docx]

| Question | n (%) |
| --- | --- |
| Physical burden on the physician is an underexposed area in the medical field  Strongly Disagree  Disagree  Neutral  Agree  Strongly Agree | 400  2 (0.5)  14 (3.5)  64 (16)  137 (34.3)  183 (45.8) |
| Ergonomics is an underexposed area in the neurosurgical field  Strongly Disagree  Disagree  Neutral  Agree  Strongly Agree | 401  4 (1)  11 (2.7)  46 (11.5)  142 (35.4)  198 (49.4) |
| Wearing a lead apron while operating increases physical discomfort  Strongly Disagree  Disagree  Neutral  Agree  Strongly Agree | 397  6 (1.5)  8 (2)  35 (8.8)  89 (22.4)  259 (65.2) |
| Neurosurgery residency training programs/curricula should be revised to provide surgical ergonomics education to trainees  Strongly Disagree  Disagree  Neutral  Agree  Strongly Agree | 401  9 (2.2)  26 (6.5)  52 (13)  137 (34.2)  177 (44.1) |
| Minimally invasive surgery leads to more physical discomfort than open surgery  Strongly Disagree  Disagree  Neutral  Agree  Strongly Agree | 397  34 (8.6)  83 (20.9)  160 (40.3)  76 (19.1)  44 (11.1) |
| Hospital management should invest more resources to equip the operating room more ergonomically  Strongly Disagree  Disagree  Neutral  Agree  Strongly Agree | 400  9 (2.3)  10 (2.5)  32 (8)  105 (26.3)  244 (61) |
| Musculoskeletal discomfort is more pronounced towards the end of the day  Strongly Disagree  Disagree  Neutral  Agree  Strongly Agree | 401  3 (0.7)  11 (2.7)  36 (9)  113 (28.2)  238 (59.4) |
| Musculoskeletal discomfort is more pronounced towards the end of the week  Strongly Disagree  Disagree  Neutral  Agree  Strongly Agree | 401  12 (3)  32 (8)  90 (22.4)  110 (27.4)  157 (39.2) |
